# Best-Compromise Control Strategy Between Mechanical Energy Expenditure and Foot Clearance for Obstacle-Crossing in Older Adults: Effects of Tai-Chi Chuan Practice

**DOI:** 10.3389/fbioe.2021.774771

**Published:** 2021-12-02

**Authors:** Chien-Chung Kuo, Sheng-Chang Chen, Jr-Yi Wang, Tsung-Jung Ho, Tung-Wu Lu

**Affiliations:** ^1^ Department of Orthopedics, China Medical University Hospital, Taichung, Taiwan; ^2^ Department of Orthopedics, School of Medicine, China Medical University, Taichung, Taiwan; ^3^ Department of Biomedical Engineering, National Taiwan University, Taipei, Taiwan; ^4^ Department of Orthopedics, Shuang Ho Hospital, Taipei Medical University, Taipei, Taiwan; ^5^ Integration Center of Traditional Chinese and Modern Medicine, Buddhist Tzu Chi General Hospital, Hualien, Taiwan; ^6^ Department of Chinese Medicine, Buddhist Tzu Chi General Hospital, Hualien, Taiwan; ^7^ School of Post-Baccalaureate Chinese Medicine, Tzu Chi University, Hualien, Taiwan

**Keywords:** gait analysis, optimal control, obstacle crossing, Tai-Chi Chuan, risk of fall

## Abstract

**Background:** Obstacle-crossing increases the risk of falls in older people. This study aimed to identify the effects of long-term Tai-Chi Chuan (TCC) practice on the control strategies for obstacle-crossing in older people.

**Methods:** A multi-objective optimal control technique with measured gait data was used to identify the control strategies adopted by 15 long-term TCC practitioners and 15 healthy controls when crossing obstacles of different heights, in terms of the best-compromise weighting sets for the conflicting objectives of minimizing energy expenditure and maximizing the toe-obstacle and heel-obstacle clearances.

**Results and Conclusions:** The long-term TCC older practitioners adopted a best-compromise control strategy similar to those adopted by young adults, with greater weightings on the minimization of the mechanical energy expenditure and smaller weightings on foot-clearance as compared to non-TCC controls (TCC: 0.72, 0.14, 0.14; Control: 0.55, 0.225, 0.225). This strategy enabled the long-term TCC older practitioners to cross obstacles with significantly greater leading-toe clearances but with relatively less mechanical energy expenditure. With the current approach, further simulations of obstacle-crossing mechanics with a given weighting set will be useful for answering clinically relevant what-if questions, such as what abilities would be needed if the non-TCC older people were to cross obstacles using the crossing strategy of the TCC people.

## Introduction

More than 25% of older adults reported falling at least once a year, with a higher incidence of falls in frail individuals or those with disabilities (Blake et al., 1988; Tinetti et al., 1988; Tinetti et al., 1994; Graafmans et al., 1996; Bergen et al., 2016). Among the causes of falls in the elderly, losing balance during obstacle-crossing is one of the most frequent (Blake et al., 1988; Gillespie et al., 2003; Tinetti, 2003). Maintaining the body’s stability, together with sufficient foot clearance of the swing limb, is essential for successful obstacle-crossing. Inappropriate control of the locomotor system may contribute to body imbalance or tripping over obstacles. Previous studies on obstacle-crossing have suggested a need for better strategies for improved balance and end-point control to reduce fall risks in the elderly (Chen et al., 1994; Chou and Draganich, 1998; Draganich and Kuo, 2004; McKenzie and Brown, 2004; Lu et al., 2006). Tai-Chi Chuan (TCC) as a low-speed, low impact exercise is beneficial for retaining or regaining balance for older people (Lai et al., 1995). However, it remains unclear whether TCC practice would help develop better movement control during obstacle-crossing in the elderly for reduced risk of falls.

The current knowledge of the mechanics of obstacle-crossing has been established primarily by using marker-based stereophotogrammetry with inverse dynamics analysis techniques to obtain the angular and moment changes at the lower limb joints when crossing obstacles of different heights in various subject populations (Liu et al., 2010; Lu et al., 2010; Chien and Lu, 2017; Chen et al., 2018; Liu et al., 2018). These data are helpful for identifying joint level alterations associated with age, pathology, or interventions. However, it is difficult to synthesize such data to uncover the overall control strategy of obstacle-crossing. Several attempts have been made over the last decades to tackle this problem using single-objective optimal control techniques without success (Chou et al., 1997; Armand et al., 1998). Previous studies have shown that normal level walking is governed by a control strategy of minimizing energy expenditure (Nubar and Contini, 1961; Dean, 1965; Beckett and Chang, 1968; Zarrugh, 1981; Chou et al., 1995) but minimizing energy expenditure alone did not predict experimentally observed motions of the leading swing ankle during obstacle-crossing (Chou et al., 1997; Lu et al., 2012). It is evident that for a successful obstacle-crossing, the swing foot has to cross over the obstacle with sufficient foot clearance (Chen and Lu, 2006). Older adults increase toe-obstacle clearance to reduce the odds of the foot hitting the obstacle and thus reduce the risk of tripping (Lu et al., 2006). Lifting the swing limb to increase the foot-obstacle clearance may increase the energy expenditure needed for the associated body postural adjustments while maintaining balance. Therefore, energy expenditure minimization and foot clearance maximization are conflicting in nature and have to be considered simultaneously during obstacle-crossing (Lu et al., 2012).

**FIGURE 1:**
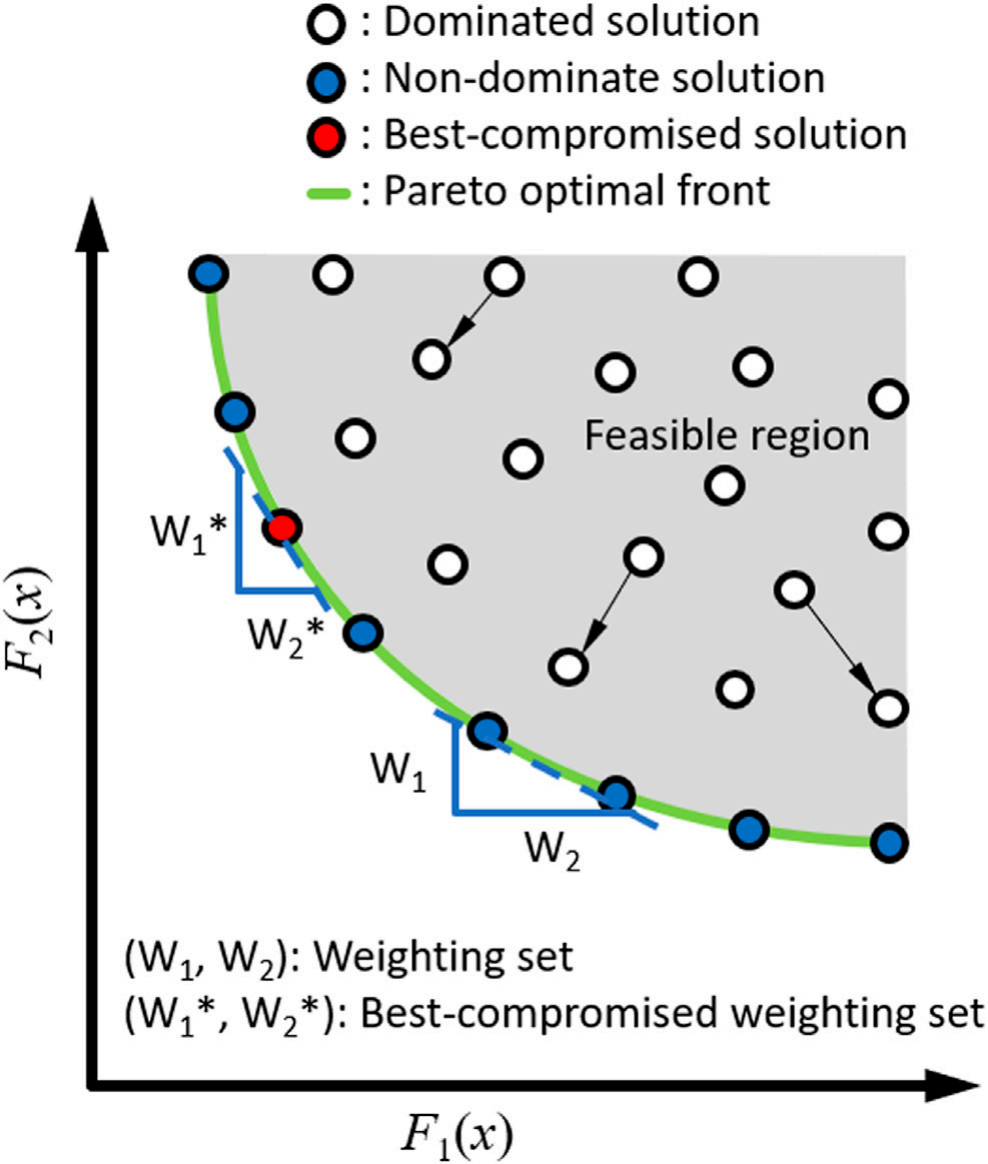
Feasible region, non-dominated (Pareto) solutions (green line) and the best-compromise solution (red circle) of a two-objective optimization problem of a single design variable *x* in the objective function domain [*F*_*1*_(*x*), *F*_*2*_(*x*)]. Within the feasible region, solutions are called dominated (e.g., white circles) if both objective functions can be improved simultaneously. In contrast, a solution is called non-dominated (filled circles) if none of the objective functions can be improved without degrading the other objective function values. All nondominated solutions are considered equally good unless a single nondominated solution called best-compromise solution is chosen according to the utility function, which defines the decision maker’s preference structure in terms of weightings associated with the objective functions (W_1_ and W_2_). The best-compromise weighting set (W_1_* and W_2_*) identifies the best-compromise solution that maximizes the decision maker’s utility function among the non-dominated solutions.

With a novel multi-objective optimal control (MOOC) approach, Lu *et al.* showed that the overall control strategy for obstacle-crossing in young adults is the best-compromise between minimization of energy expenditure and maximization of foot-obstacle clearance and that the MOOC strategy is stored and executed in the central control system (Lu et al., 2012). For a multi-objective optimization problem with conflicting objective functions, there exists a (possibly infinite) number of nondominated (Pareto) optimal solutions (Figure 1). A solution is called nondominated if none of the objective functions can be improved in value without degrading other objective function values (Tseng and Lu, 1990). All nondominated solutions are considered equally good unless a single nondominated solution called best-compromise solution is chosen according to the utility function, which defines the decision maker’s preference structure in terms of weightings associated with the objective functions. The best-compromise weighting set identifies the best-compromise solution that maximizes the decision maker’s utility function among the non-dominated solutions, and can be obtained using a weighting method (Tseng and Lu, 1990). In obstacle-crossing, Lu et al. showed that the solution with a weighting set of (0.68; 0.16; 0.16) for the minimization of energy expenditure and maximization of heel-obstacle and toe-obstacle clearances predicts the swing ankle trajectories, joint angles, and moments accurately during obstacle-crossing in young adults (Lu et al., 2012). These results indicate that the solution is the best-compromise solution chosen by the central nervous system (CNS) as a decision-maker for the MOOC problem of obstacle-crossing in the young (McFadyen, 1991; Maclellan and McFadyen, 2010; Haefeli et al., 2011; Lu et al., 2012; MacLellan, 2017). With aging, there is a natural degradation of the functions of the neuromusculoskeletal system, leading to joint kinematic and kinetic changes during obstacle-crossing. A recent study also showed that aging affects the MOOC control strategy of obstacle-crossing (Kuo et al., 2021). Older adults adopted a crossing strategy that emphasizes foot-obstacle clearance with greater weightings to reduce the risk of tripping over the obstacle at the expense of increased energy expenditure with smaller weighting (Kuo et al., 2021). This strategy indicates that it is essential to maintain or improve muscle strength and limb position control abilities for safe and successful obstacle-crossing in the older population. The MOOC approach is also expected to help for evaluating the efficacy of interventions aiming at reducing the risks of falls in older people during obstacle-crossing.

Tai-Chi Chuan (TCC) is an ancient Chinese martial art that has become a popular exercise for improving general mental and physical fitness, especially in the older population (Penn et al., 2019; Xiao et al., 2020). From the physical exercise viewpoint, TCC focuses on dynamic weight-shifting when transitioning from double-limb to single-limb support during a series of slow, continuous movements with well-controlled body posture (Wu et al., 2004). Older people have been shown to benefit from TCC practice in many aspects, including critical components for preventing falls (Tse and Bailey, 1992; Wolfson et al., 1996; Wolf et al., 1997a; Hass et al., 2004) such as muscle strength (Jacobson et al., 1997; Lan et al., 2000; Wu et al., 2002), body flexibility (Lan et al., 1996; Lan et al., 1998), sensory organization in postural and balance control (Wong et al., 2001; Wu, 2002; Wu et al., 2002; Mak and Ng, 2003; Tsang et al., 2004). From the limited studies in the literature, long-term TCC practice appeared to help attenuate the age-related decline in physical fitness in older people, improving their gait patterns and abilities in movement modifications during obstacle-crossing (Ramachandran et al., 2007; Chang et al., 2015). In such cases, older people with long-term TCC experience may maintain a MOOC strategy for obstacle-crossing similar to young adults. However, no study has reported whether long-term TCC practice positively affects the MOOC control strategy of obstacle-crossing in older adults.

The purpose of the current study was to identify the optimal control strategies adopted by healthy older people and long-term TCC practitioners when crossing obstacles of different heights with the leading limb using a MOOC technique with a mechanical model of the human body in the sagittal plane. It was hypothesized that, compared to non-TCC healthy older people, long-term TCC practitioners would show a different MOOC strategy for obstacle-crossing with greater weighting to the minimization of energy expenditure, which would enable them to cross obstacles of different heights with greater leading-toe clearances but with relatively less mechanical energy expenditure.

## Materials and Methods

### Subjects

Fifteen healthy older adults who practiced TCC at least 60 min a day and 5 days a week for thirteen or more years (TCC group, gender: 4 females/11 males, age: 71 ± 5.4 years, height: 163 ± 6.7 cm, body mass: 59 ± 6.5 kg, TCC experience: 22 ± 10.5 years) participated in the current study with informed written consent as approved by the Institutional Review Board (IRB No. DMR98-IRB-072). Fifteen healthy controls without TCC experience but doing daily walking or jogging (Control group, gender: 4 females/11 males, age: 72 ± 6.2 years, height: 160 ± 5.6 cm, mass: 58 ± 10.5 kg) were also recruited to match with the TCC group for gender, age and BMI with informed written consent. All the subjects had a normal or corrected vision. None of the subjects had any neuromusculoskeletal disease or dysfunction that might affect gait and obstacle-crossing. An *a priori* power analysis based on data from a previous study (Kuo et al., 2021) using G*POWER (Erdfelder et al., 1996) for two-way mixed-design analysis of variance (ANOVA) determined that a projected sample size of 14 subjects for each group would be needed with a power of 0.8 and a large effect size (Cohen’s d = 0.69) at a significance level of 0.05. Thus, 15 subjects for each group were considered adequate for the main objectives of the current study.

### Gait Experiment

In a gait laboratory, each subject wearing 39 infrared-retroreflective markers on specific anatomical landmarks of the body segments (Hong et al., 2015) walked on a 8-m walkway at their preferred walking speed and crossed an obstacle located in the middle of the walkway (Chen et al., 2008; Hsu et al., 2010). The obstacle was made of an aluminum tube placed horizontally across a height-adjustable metal frame. A seven-camera motion capture system (Vicon 512, Oxford Metrics Group, United Kingdom) was used to measure the three-dimensional trajectories of the body-worn markers at 120 Hz for the definition of the poses of the body segments. Two infrared-retroreflective markers placed on each end of the tube were also used to define the position and height of the obstacle. Two forceplates (AMTI, United States) placed on either side of the obstacle were used to measure the ground reaction forces (GRF) and the center of pressure (COP) at 1,080 Hz. The test conditions were crossing obstacles of 10, 20, and 30% of the subject’s leg length (i.e., distance between the ASIS and medial malleolus) in a random order, with each lower limb leading (Chen and Lu, 2006). A trial was defined as unsuccessful if the subject hit the obstacle during the crossing. In the current study, all the subjects could cross the obstacles successfully without hitting the obstacle. Data for three successful crossing trials for each lower limb leading were obtained for each obstacle height for each subject. A 5-min break was allowed when changing obstacle-height conditions.

### Human Body Model

**FIGURE 2:**
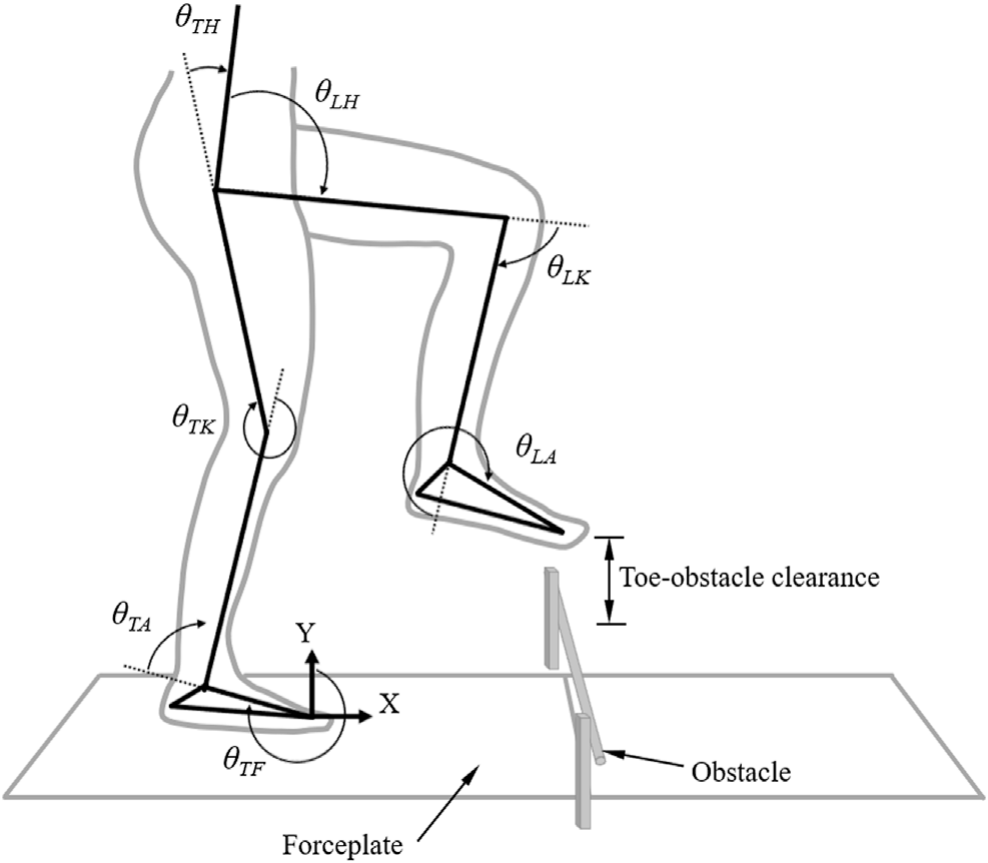
The planar seven-link model of the body when obstacle-crossing. Two forceplates are placed on either side of the obstacle. Definitions of the toe-obstacle clearance and the joint angles are also indicated: hip ( $\theta_{LH}$ ), knee ( $\theta_{LK}$ ) and ankle ( $\theta_{LA}$ ) of the leading swing limb, and foot ( $\theta_{TF}$ ), ankle ( $\theta_{TA}$ ), knee ( $\theta_{TK}$ ) and hip ( $\theta_{TH}$ ) of the trailing stance limb. The upper body, namely the segments of the head/neck, trunk, pelvis, and upper extremities, was modeled as a single link, defined as the line connecting the hip joint center and the center of mass of all the upper body segments. The hip angles were defined as the angles between the upper body link and the thigh links. The X-axis indicates the direction of progression.

For the computer simulations of the control of obstacle-crossing, the human body was modeled as a seven-link system in the sagittal plane, consisting of the upper body and the thighs, shanks, and feet that were connected by model hinge joints of the hips, knees, and ankles (Figure 2) (Lu et al., 2012). The upper body, namely the segments of the head/neck, trunk, pelvis, and upper extremities, was modeled as a single link, defined as the line connecting the hip joint center and the center of mass of all the upper body segments, similar to the model by Chou et al. (Chou et al., 1997). Thus, the model had seven degrees of freedom, described by seven angular displacements of the ground/foot ( $\theta_{TF}$ ), ankle ( $\theta_{TA}$ ), knee ( $\theta_{TK}$ ), and hip ( $\theta_{TH}$ ) of the trailing stance limb, and the hip ( $\theta_{LH}$ ), knee ( $\theta_{LK}$ ), and ankle ( $\theta_{LA}$ ) of the leading swing limb (Figure 2). The hip angles were defined as the angles between the upper body link and the thigh links. The foot of the trailing stance limb was assumed to be connected to the ground at the ground/foot joint ( $\theta_{TF}$ ), so the reaction forces and moments at the hinge joint were equivalent to the measured GRF. Subject-specific model parameters, namely lengths and inertial properties of the links and joint center positions were determined using a model-based optimization method that minimizes errors between model-predicted and measured COP positions during several calibration postures (Chen et al., 2011). The equations of motion governing the dynamics of the seven-link model are given as follows (Lu et al., 2012):

$$M\left(\theta \right)\ddot{\theta }=T\left(t\right)+V\left(\theta \right){\dot{\theta }}^{2}+G\left(\theta \right)+E(\theta ,\dot{\theta }),$$ (1)

where θ , $\dot{\theta }$ , $\ddot{\theta }$ are 7×1 vectors of joint angular displacements, velocities, and accelerations, respectively, M(θ) is a 7×7 mass matrix, T(t) is a 7×1 vector of joint moments, $V\left(\theta \right){\dot{\theta }}^{2}$ is a 7×1 vector describing both Coriolis and centrifugal effects, G(θ) is a 7×1 vector of gravitational forces, and $E\left(\theta ,\dot{\theta }\right)$ is a 7×1 vector of external forces (Lu et al., 2012).

### Multi-Objective Optimal Control Problem

In the current control study of obstacle-crossing, an inverse dynamics approach was adopted for the calculation of the joint moments. The leading swing ankle trajectory was taken as control variable while the joint angles of the trailing stance limb joints were prescribed using subject-specific experimental data (Chou et al., 1995). This simplification was based on the fact that the trailing stance limb joints experience much smaller ranges of motion than those of the leading swing limb (Chou et al., 1995; Chen et al., 2004). Therefore, the experimentally measured input data for the MOOC of obstacle-crossing are the initial and final positions of the ankle of the leading swing limb at toe-off immediately before crossing ( $t_{1}$ ) and at subsequent initial contact ( $t_{n}$ ), the angular histories of the swing ankle ( $\theta_{LA}$ ), and the angular histories of the foot ( $\theta_{TF}$ ), ankle ( $\theta_{TA}$ ), knee ( $\theta_{TK}$ ), and hip ( $\theta_{TH}$ ) of the stance limb (Figure 2). Given the input data, there were an infinite number of leading swing ankle trajectories that defined the angular trajectories of the leading swing hip ( $\theta_{LH}$ ) and knee ( $\theta_{LK}$ ) considering the linkage of the leading swing limb (Figure 2). Therefore, the leading swing ankle trajectory was the only variable to be controlled for the inverse dynamic analysis of obstacle-crossing, giving time histories of the angles of the leading swing hip and knee, and the joint moments of both the stance and swing limbs. According to Lu *et al.* (Lu et al., 2012) the control of the lower limb during obstacle-crossing is a trade-off between minimization of mechanical energy and maximization of swing heel-obstacle and toe-obstacle clearances. Therefore, the MOOC problem of obstacle-crossing became the search for the positional trajectory of the swing ankle that minimized the mechanical energy expenditure ( $f_{1}$ ) and maximized the clearances of the swing heel ( $f_{2}$ ) and toe ( $f_{3}$ ) above the obstacle. The MOOC problem was converted to a non-linear programming (NLP) problem via a parameterization approach which discretized the spatial trajectory of the swing ankle to *n* spatial positions at equal-timed intervals ( $\Delta t=\frac{t_{n}-t_{1}}{n}$ ) over the total time of the swing phase, which were taken as the design variables of the NLP problem, i.e., $\;\bar{y}=\left(y_{1},y_{2},...,y_{n}\right)$ . Since a large *n*-value increases the dimensionality and the computational effort for solving the NLP problem without necessarily improving the accuracy, an *n*-value of 20 was selected based on previous numerical experience (Lu et al., 2012). Given a set of values for the design variables, the model calculated the joint angular displacements at the *n* instants, which were then fitted with quintic splines and differentiated once and twice to obtain angular velocities and accelerations respectively for each joint and segment using the GCVSPL method (Woltring, 19781986). With these information, joint moments were then calculated using inverse dynamics analysis with Eq. 1. The parameterized MOOC problem can be described as follows:

Find a set of design variables, $\bar{y}=\left(y_{1},y_{2},...,y_{n}\right)$ to minimize the following objective functions

$$f_{1}\left(\bar{y}\right)=\sum_{j=1}^{7}\sum_{i=1}^{n}T_{j}^{i}\omega_{j}^{i}\Delta t,$$ (2)

$$f_{2}\left(\bar{y}\right)=-d_{h},$$ (3)

$$f_{3}\left(\bar{y}\right)=-d_{t},$$ (4)

subject to the equations of motion of the seven-link model defined in Eq. 1. Mechanical energy expenditure ( $f_{1}$ ) was calculated as the summation of the products of the joint moments ( $T_{j}^{i}$ ), joint angular velocities ( $\omega_{j}^{i}$ ), and time interval Δt for all joints and time intervals where i indicated the *i*th time instant and j indicated the *j*th model joint. The terms $d_{t}$ and $d_{h}$ were the toe-obstacle and heel-obstacle clearances, respectively, when the swing toe and heel were above the obstacle.

### Multi-Objective Optimal Control Solution Using Weighting Method

The MOOC problem was solved using the weighting method (Tseng and Lu, 1990), which converted the problem into a single objective minimization problem with the weighted sum of the original objectives as the new objective function, i.e., ( $W_{1}f_{1}+W_{2}f_{2}+W_{3}f_{3}$ ). $W_{1}$ , $W_{2}$ , and $W_{3}$ were the weighting factors for the mechanical energy expenditure ( $f_{1}$ ), heel-obstacle clearance ( $f_{2}$ ) and toe-obstacle clearance ( $f_{3}$ ), respectively, and satisfying $W_{1}+W_{2}+W_{3}=1$ . For a set of prescribed weighting factors, the converted MOOC problem was to find a set of design variables, $\bar{y}=\left(y_{1},y_{2},...,y_{n}\right)$ , to minimize the following objective function

$$f\left(\bar{y}\right)=W_{1}\frac{f_{1}\left(\bar{y}\right)}{f_{1}^{*}}+W_{2}\frac{f_{2}\left(\bar{y}\right)}{f_{2}^{*}}+W_{3}\frac{f_{3}\left(\bar{y}\right)}{f_{3}^{*}}$$ (5)

where $f_{1}^{*}$ , $f_{2}^{*}$ and $f_{3}^{*}$ were the optimum values of the NLP problem with each of the objective functions separately as the single objective. Normalization of the objective functions by the corresponding optimum values helped eliminate the effects of the differences in the units of the objective functions. Energy expenditure was also normalized to the movement duration. The best-compromise solution to the original MOOC problem was obtained as the optimum solution to the converted MOOC problem in Eq. 5 with a set of weighting factors ( $W_{1}^{*},\;W_{2}^{*},\;W_{3}^{*}$ ) that gave minimum root mean squared errors (RMSEs) between the measured and model-predicted ankle trajectories. For each subject and each test condition, the associated MOOC problem was solved using an in-house-developed program in MATLAB (Math Works, United States).

In order to obtain input data for the 2D MOOC analysis and subsequent comparisons between MOOC best-compromise results and experimental measurements, the center of mass of all the upper body segments and the joint centers, angles, and moments of the lower limbs were calculated from experimental data using inverse dynamics analysis with a validated 3D model of the locomotor system (Lu and O'Connor, 1998), and then projected onto the sagittal plane. The RMSEs between the measured and best-compromise trajectories of the swing ankle joint center and joint angles of the leading limb and joint moments of both lower limbs were calculated for all height conditions and each subject.

### Statistical Analysis

For statistical analysis, clearances and distances were normalized to the subject’s leg length, and mechanical energy expenditure was normalized to body weight, leg length, and the total time of the crossing swing phase. Such normalization was helpful for reducing the effects of the relevant variabilities among individual subjects. Similar normalization approaches are widely used in the area of human motion analysis (Chou et al., 2001; Chen et al., 2004; Kuo et al., 2021). The crossing speed, end-point variables, energy expenditure, best-compromise weighting sets, and the RMSEs of the calculated ankle positions and joint angles of the leading swing limb, and the joint moments of the trailing stance limb were analyzed using a two-way mixed-design analysis of variance (ANOVA) with one between-subject factor (group) and one within-subject factor (obstacle height). If there was no interaction, main effects were reported. Otherwise, pair-wise between-group comparisons were performed using an independent *t*-test for each obstacle height, and a *post-hoc* trend analysis was performed to determine the trend of the variable with increasing obstacle height for each group. All significance levels were set at α = 0.05. SPSS version 20 (SPSS Inc., Chicago, United States) was used for all statistical analyses.

## Results

**FIGURE 3:**
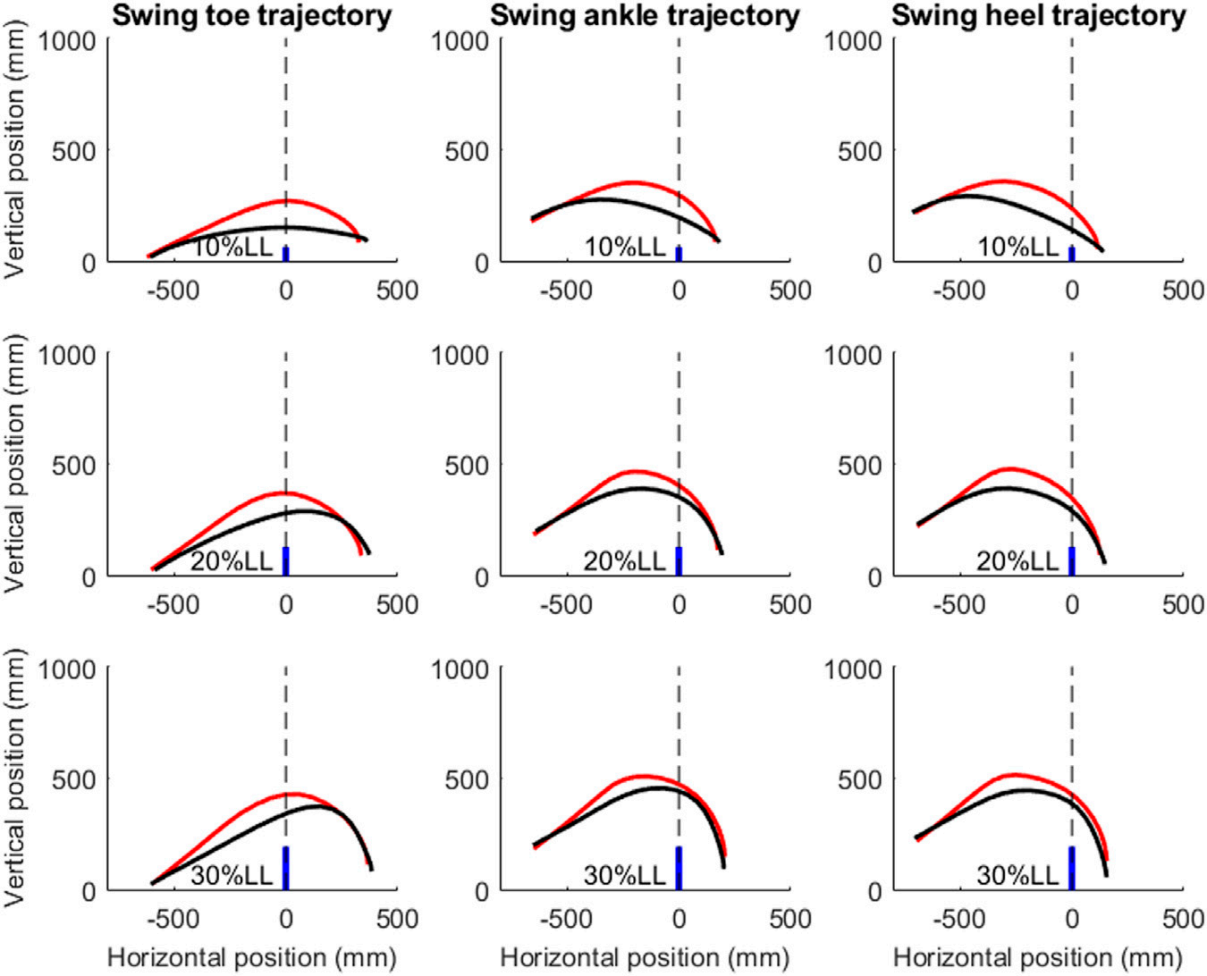
Sagittal trajectories of the toe, ankle, toe, and heel of the leading swing limb of a typical subject for each of the TCC (red curves) and control (black curves) groups when crossing obstacles of 10, 20, and 30% of the subject’s leg length (LL) (solid blue lines).

There were no statistical interactions between group and obstacle height factors for any tested variables, so only the main effects are reported here. The TCC group showed significantly greater leading-toe, leading-heel, and trailing-toe clearances but smaller energy expenditure when compared to the Control group (Table 1 and Figure 3). With increasing obstacle height, the leading-toe, leading-heel, trailing-heel clearances, and energy expenditure were significantly increased linearly, but the crossing speed and leading heel-obstacle distances were decreased linearly (Table 1).

**TABLE 1:** Means (standard deviations) of the end-point variables for the TCC and Control groups when crossing obstacles of three different heights.

	Obstacle height (% LL)			Group effect	Height effect
	10	20	30	*p*-value	
Crossing speed (m/s)					
TCC	0.70 (0.06)	0.66 (0.7.7)	0.62 (0.07)	0.17	0.01↓
Control	0.80 (0.14)	0.70 (0.12)	0.64 (0.11)		
Leading-toe clearance (mm)					
TCC	183.1 (23.4)	195.5 (23.9)	205.9 (24.4)	0.03*	0.02↑
Control	156.1 (34.3)	168.8 (32.6)	175.5 (40.3)		
Trailing-toe clearance (mm)					
TCC	164.5 (30.4)	179.3 (43.1)	173.9 (36.5)	0.03*	0.48
Control	131.1 (44.2)	134.5 (49.8)	145.4 (54.9)		
Leading-heel clearance (mm)					
TCC	147.0 (39.2)	153.2 (35.1)	172.2 (31.5)	0.04*	0.01↑
Control	120.4 (24.5)	133.7 (24.3)	141.4 (31.8)		
Trailing-heel clearance (mm)					
TCC	340.8 (50.0)	361.2 (45.1)	361.7 (44.6)	0.72	0.04↑
Control	335.1 (48.5)	344.9 (49.3)	365.8 (73.0)		
Leading heel-obstacle distance (% LL)					
TCC	17.33 (2.8)	16.43 (3.3)	15.59 (3.5)	0.63	0.01↓
Control	19.63 (4.3)	16.30 (3.7)	15.20 (4.6)		
Trailing toe-obstacle distance (% LL)					
TCC	24.94 (4.8)	25.35 (4.8)	24.38 (5.1)	0.86	0.28
Control	24.94 (4.4)	24.60 (4.2)	24.24 (4.8)		
Energy expenditure (% body weight×LL×total time)					
TCC	143.1 (22.3)	148.0 (24.8)	157.4 (26.0)	0.04*	0.01↑
Control	170.4 (46.9)	169.7 (33.3)	182.7 (37.9)		

LL: leg length; *: significant difference between subject groups; ↑: linearly increasing trend; ↓: linearly decreasing trend; Total time: total time of the crossing swing phase

**FIGURE 4:**
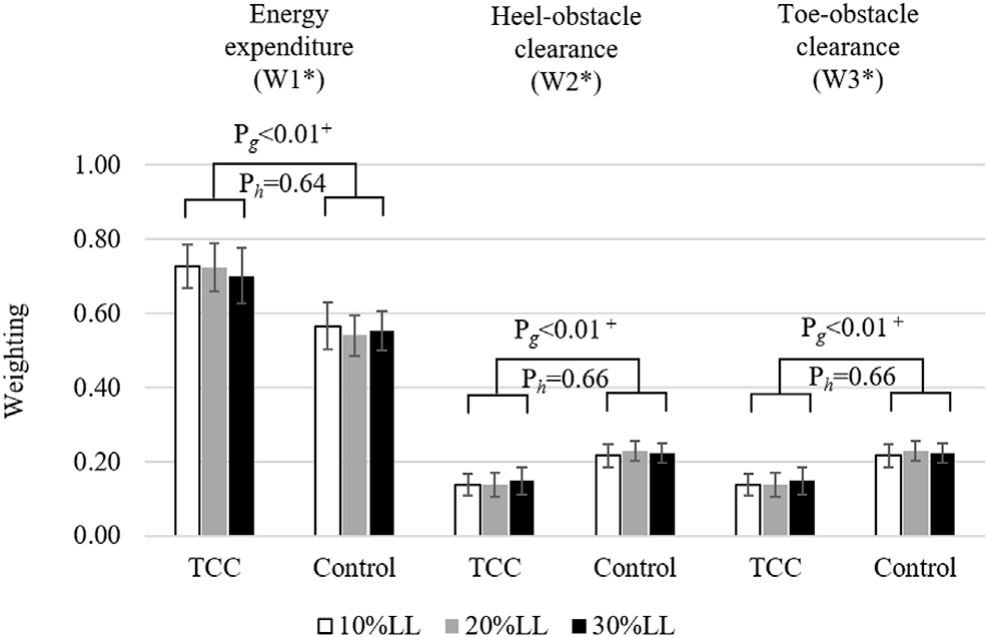
Means (standard deviations) of the best-compromise weighting sets (W_1_*, W_2_*, W_3_*), corresponding to the objective functions of mechanical energy expenditure, heel-obstacle clearance, and toe-obstacle clearance of the MOOC problem, for the TCC and Control groups when crossing obstacles of three different heights: 10% (white), 20% (grey) and 30% (black) of individual subject’s leg length (LL). *P*-values for the main effects are also given (P_g_: group effects; P_h_: height effects). +: statistically significant differences between groups (*p* < 0.05).

The TCC group showed significantly increased mechanical energy expenditure weightings but decreased weightings to the heel- and toe-clearances (W1: 0.72; W2: 0.14; W3: 0.14) when compared to the Control group (W1: 0.55; W2: 0.225; W3: 0.225) (*p* < 0.01, Figure 4). No significant height effects were found for the best-compromise weighting sets (*p* > 0.05, Figure 4).

With the best-compromise weighting sets, the RMSEs of the leading ankle trajectories over the swing phase between the MOOC best-compromise results and experimental measurements were less than 1.6 mm for all obstacle heights and for both groups without significant group or height effects (*p* > 0.05, Table 2). Similarly, the RMSEs for the swing hip and knee angles were less than 0.7° for all obstacle heights and for both groups without significant group or height effects (Table 2). The RMSEs for the joint moments of the trailing stance limb were less than about 6.7 Nm for both groups and all obstacle heights without significant group or height effects (Table 2).

**TABLE 2:** Means (standard deviations) of the RMSEs of the ankle trajectories and joint angles of the leading limb, and the joint moments of the trailing limb obtained by the MOOC with the best-compromise weighting set against the experimental data in the TCC and Control groups.

	Obstacle height (% LL)			Group effect	Height effect
	10	20	30	*p*-value	
Ankle trajectory (mm)					
TCC	1.60 (0.55)	1.56 (0.51)	1.26 (0.38)	0.82	0.28
Control	1.42 (0.45)	1.60 (0.62)	1.51 (0.38)		
Swing hip angle (°)					
TCC	0.48 (0.25)	0.38 (0.11)	0.32 (0.10)	0.12	0.41
Control	0.29 (0.13)	0.24 (0.15)	0.37 (0.35)		
Swing knee angle (°)					
TCC	0.63 (0.14)	0.58 (0.18)	0.51 (0.19)	0.17	0.22
Control	0.53 (0.19)	0.47 (0.19)	0.47 (0.24)		
Trailing ankle moment (Nm)					
TCC	4.53 (6.32)	6.05 (7.45)	5.31 (6.06)	0.90	0.30
Control	4.34 (2.42)	4.09 (1.87)	6.62 (7.40)		
Trailing knee moment (Nm)					
TCC	3.68 (4.56)	4.31 (5.03)	3.90 (4.74)	0.96	0.73
Control	3.60 (3.10)	3.13 (1.72)	5.38 (3.25)		
Trailing hip moment (Nm)					
TCC	3.23 (3.21)	4.02 (3.28)	3.39 (2.89)	0.89	0.39
Control	3.57 (2.76)	2.99 (1.74)	4.55 (2.88)		

## Discussion

The current study aimed to identify the effects of long-term TCC practice on the control strategies adopted by healthy older people when crossing obstacles of different heights using a MOOC technique with a mechanical model of the human body in the sagittal plane. The long-term TCC older practitioners were found to adopt a best-compromise control strategy for obstacle-crossing similar to those adopted by young adults, with a greater weighting on the minimization of the mechanical energy expenditure and smaller weightings on foot-clearance as compared to non-TCC controls. This strategy enabled the long-term TCC older practitioners to cross obstacles with significantly greater leading-toe clearances but less mechanical energy expenditure than non-TCC controls. Both groups showed control strategies and end-point variables independent of obstacle height, suggesting that the two different control strategies were at the central nervous system level.

The MOOC approach provides a useful platform for identifying the control strategies used during obstacle-crossing from a system level perspective and for comparisons between different subject populations. This is in contrast to most previous studies that focused on changes of state variables of individual joints, such as joint angles and moments. While state variables describe the changes of the neuromusculoskeletal system during the motor task, it is difficult to deduce the overall control principle of the system from the changes of the state variables. This is because the changes of the state variables vary with parameters of the system and the task, such as body stature and obstacle height (Sparrow et al., 1996; Begg et al., 1998). For example, some state variables changed with increasing obstacle height while others remained unaltered, and such changes vary in different studies (Chou and Draganich, 1997; Austin et al., 1999; Chen and Lu, 2006). Therefore, controversies exist in the literature regarding the conclusions derived from state variable changes, such as the effects of obstacle height on the lower limb kinematics and kinetics (Chen et al., 2004; Lu et al., 2006). However, the individual joint variables did not work independently as they appeared. The patterns of the inter-joint coordination between the lower limb joints were found to be independent of obstacle height (Lu et al., 2008; Wang et al., 2009; Yen et al., 2009). The current MOOC approach further identified the overall control strategy underlying the measured changes of the kinematic and kinetic joint variables during obstacle-crossing for both older people with and without TCC experience, confirmed by the small RMSEs of the simulation results, including leading swing ankle trajectories, leading swing hip and knee angles, and trailing limb joint moments (Table 2).

The observed control strategies adopted by the current older groups for obstacle-crossing involved multiple objectives, i.e., minimization of mechanical energy expenditure and maximization of foot-obstacle clearances, in agreement with the previous study on young adults (Lu et al., 2012). It is fundamentally different from the single-objective control strategy governing unobstructed level walking, i.e., minimization of mechanical energy expenditure. Through the systematic search over the nondominated solutions (or Pareto solutions) using the weighting method, the nondominated solution that produced results best matched the subject-specific experimental data, i.e., the best-compromise solution, was found for each of the subjects. The neuromusculoskeletal systems of the TCC and Control groups showed different preferences on the individual objectives, giving different best-compromise solutions between the conflicting objectives of minimizing energy and maximizing foot clearance. In the best-compromise control strategy, the mean weightings for mechanical energy expenditure were 0.72 for TCC and 0.55 for Control in the current study, and 0.68 for young adults in the literature (Lu et al., 2012). These results show how the different groups of people modified their control strategies differently from the minimization of energy expenditure for unobstructed level walking (energy weighting: 1.0) to negotiate with the obstacles of different heights, reflecting different physical conditions of the subject groups. This suggests that obstacle-crossing is a motor task suitable for the evaluation of the overall performance of the neuromusculoskeletal system between populations or after interventions such as TCC training.

The long-term TCC older practitioners were able to adopt a single control strategy for obstacle-crossing of different heights similar to those adopted by young adults (Lu et al., 2012), with greater weightings on the minimization of the mechanical energy expenditure and smaller weightings on foot-clearance as compared to non-TCC controls. This strategy enabled the long-term TCC older practitioners to cross obstacles with significantly greater leading-toe clearances for reduced risk of tripping but with relatively less mechanical energy expenditure than older peers. These results suggest that the long-term TCC practitioners had the necessary muscle strength and ability of precision limb position control to carry out a strategy similar to that used by the young for obstacle-crossing (Tse and Bailey, 1992; Wolfson et al., 1996; Wolf et al., 1997b; Hass et al., 2004). Previous studies have shown that training through TCC movements improved muscle strength (Wu et al., 2002), body flexibility (Lan et al., 1996; Lan et al., 1998) and sensory organization in postural and balance control (Wong et al., 2001; Wu, 2002; Wu et al., 2002; Mak and Ng, 2003; Tsang et al., 2004). In TCC training, the sum of the percentage duration of fixing, forward, and backward movements was about 60%, and that of single-limb support was almost 70% (Hong et al., 2008), which were all helpful for increasing the muscle strength in the lower extremities and improving whole-body balance (Jacobson et al., 1997; Wu et al., 2002; Ho et al., 2012). However, improved muscle strength and balance do not necessarily lead to the specific control strategy.

Long-term TCC older practice appeared helpful for forming the specific control strategy for obstacle-crossing at the central control nervous system level. The MOOC strategy found in the current study was independent of obstacle-height, suggesting a motor program stored and executed in the central nervous system (Grillner and Wallén, 1985). According to the central pattern generator theory, a motor program is an abstract representation of a movement that organizes and controls the many degrees of freedom of the body involved in performing the movement (Schmidt et al., 2018). The motor program is considered generalized because different program parameters will give different outputs. Whenever the program is executed with a particular set of parameters, the movement will be generated with a unique pattern of states of the degrees of freedom (Schmidt et al., 2018). The current findings suggest that the same motor program controlled obstacle-crossing in response to different obstacle heights (parameters). From the current results and previous studies on healthy young and older adults (Lu et al., 2012; Kuo et al., 2021), it appeared that the motor programs for obstacle-crossing are affected differently by aging and TCC training. Long-term TCC training changed the way the older people crossed obstacles of different heights with reduced risk of tripping not only by improving their muscle strength and balance control (Low et al., 2017; Hosseini et al., 2018) but also by forming a MOOC strategy similar to that used by young adults (Lu et al., 2012). This particular motor program would generate a unique pattern of states of the degrees of freedom of the whole body in performing the task. Further study on the resulting motions of the body’s center of mass relative to the base of support of the trailing stance limb may be helpful for the identification of the connections between the control strategy and the state changes of the degrees of freedom associated with TCC training.

In the current study, mechanical energy expenditure was considered in the MOOC problem. Metabolic energy expenditure was not included, as it is often measured experimentally and difficult if not impossible to model. Nonetheless, according to Burdett et al. (Burdett et al., 1983), there are strong linear relationships between metabolic and mechanical energy expenditure for the same motor task. Therefore, mechanical energy expenditure may be considered an indication of metabolomic energy expenditure for the current study on obstacle-crossing. During walking, raising the limb to cross obstacles would increase the metabolic and mechanical energy expenditure, which would increase with increasing obstacle height (Table 1). Increasing the leading toe-obstacle clearance would also increase the mechanical energy expenditure (Table 1). These results further confirmed the conflicting nature between minimization of mechanical energy expenditure and maximization of toe-obstacle clearance within a subject group. Compared to the Control group, the TCC group showed reduced mechanical energy expenditure but increased toe-obstacle clearance for all obstacle heights, suggesting that long-term TCC training helped older people cross obstacles of different heights with better efficiency. Given the between-group differences in the individual variables of mechanical energy expenditure and leading toe-obstacle clearance, the MOOC approach further showed the changes of control strategies in the TCC group, placing greater emphasis on minimizing mechanical energy expenditure than increasing foot-obstacle clearance. Note that the weightings are relative importance between the objective functions within a person or a subject group, which do not indicate the absolute cost function values between groups.

The current study was the first in the literature to reveal the overall control strategy of obstacle-crossing in older people with long-term experience of practicing TCC. The observed change in the best-compromise weighting sets with TCC training provides a synthesized explanation to the changes of the state variables across all the obstacle heights compared to older people without TCC experience. Several assumptions were made to simplify the modeling so that the analysis became feasible, including modeling in the sagittal plane, considering the upper body as a rigid body, and prescribing joint angles to the stance limb. However, these were also limitations of the study. Further studies will be needed to evaluate the possible alterations in the best-compromise weighting sets when some assumptions are released. For example, further inclusion of frontal and transverse plane components will be necessary, especially for populations with neuromusculoskeletal disorders with greater motions out of the sagittal plane (Patla and Prentice, 1995; Chou et al., 2003; Vimercati et al., 2012). In the current study, sagittal plane simulations were acceptable because normal obstacle-crossing occurred mainly in this plane (Chou et al., 1997). The ankle trajectories and joint angles of the leading limb and the joint moments of the trailing limb in the sagittal plane were also accurately predicted by the current 2D model. Nonetheless, it is acknowledged that compensatory joint movement adjustments in the other two planes were not taken into consideration in the current study.

Modeling the upper body as a single link defined by experimentally measured motions of the associated body segments and prescribing the joint angles of the stance limb as did in previous studies (Chou et al., 1995; Chou et al., 1997; Lu et al., 2012) were considered acceptable for the current study. However, further modification of the current approach may be needed if one wishes to identify the roles of individual segments of the upper body and the trailing stance limb on the control of obstacle-crossing, especially when applied to people with altered motion patterns such as owing to pathology or aging (Krebs et al., 1992; Marigold et al., 2003; Vimercati et al., 2012). On the other hand, the current study was limited to the assessment of optimal control strategies during leading limb crossing; further studies will be needed to test whether similar results also apply to obstacle-crossing with the trailing limb. The possible effects of gender on the control strategies are a direction for further study too. With the current MOOC approach, further simulations of obstacle-crossing mechanics with targeted weighting sets will be helpful for answering clinically relevant what-if questions, such as what abilities would be needed if the non-TCC older people were to cross obstacles using the crossing strategy of the TCC people.

## Conclusion

The long-term TCC older practitioners were found to adopt a best-compromise control strategy for obstacle-crossing similar to those adopted by young adults, with greater weightings on the minimization of the mechanical energy expenditure and smaller weightings on foot-clearance as compared to non-TCC controls. This strategy enabled the long-term TCC older practitioners to cross obstacles with significantly greater leading-toe clearances but with relatively less mechanical energy expenditure. Both groups showed control strategies and end-point variables independent of obstacle height, suggesting that the two control strategies were at the central nervous system level. It appears that long-term TCC training changed the way the older people crossed obstacles of different heights with reduced risk of tripping-related falls not only by improving their muscle strength and balance control but also by forming a multi-objective optimal control strategy similar to that used the young adults. With the current MOOC approach, further simulations of obstacle-crossing mechanics with targeted weighting sets will be helpful for answering clinically relevant what-if questions, such as what abilities would be needed if the non-TCC older people were to cross obstacles using the crossing strategy of the TCC people.

## Data Availability

The original contributions presented in the study are included in the article/Supplementary Material, further inquiries can be directed to the corresponding author.
